# Applying the Rules of Evidence to Expert Testimony About Risk

**DOI:** 10.1002/bsl.70068

**Published:** 2026-05-16

**Authors:** Christopher Slobogin

**Affiliations:** ^1^ Vanderbilt University Nashville Tennessee USA

## Abstract

Expert opinion about dangerousness or risk is common at sentencing, criminal commitment proceedings and some types of pretrial detention hearings. This article argues that such evidence must be (1) “material” (logically relevant, empirically generalizable, and epistemologically germane), (2) “probative” (a measure of accuracy, which is heightened when the evidence is from an expert), (3) helpful to the factfinder (through promoting “incremental validity”), and (4) presented in a non‐prejudicial manner (i.e., in a way that minimizes the possibility it will be misused or misinterpreted).

## 1 Introduction

Proving the future is always difficult. It is especially so with respect to future offending, where it can easily fall prey to miscalculation, bias and quackery (Otero 2014). As a means of minimizing these problems, this article argues that if the law insists on asking experts whether particular criminal defendants are “dangerous” or “high risk”—which it routinely does at sentencing, during bail hearings and in civil and criminal commitment proceedings—the rules of evidence can and should govern when expert testimony on the issue should be heard.

More specifically, building on previous arguments (Slobogin 2007), I argue that, to be admissible, expert testimony about risk must be (1) “material” (logically relevant, empirically generalizable, and epistemologically germane), (2) “probative” (a measure of accuracy, which is heightened when the evidence is from an expert), (3) helpful to the factfinder (through promoting “incremental validity”), and (4) presented in a non‐prejudicial manner (i.e., in a way that minimizes the possibility it will be misused or misinterpreted). While these guidelines are derived from American law, they are similar to those followed in many other common law countries (Roach 2009). Even in countries that do not have such rules, they can spur thinking about the limits that should be imposed on expert testimony about risk.

## 2 Application of the Rules of Evidence

The assertion that the rules of evidence should govern expert testimony about risk is admittedly controversial. In the United States, the evidence rules that routinely apply at trial are often ignored in the pretrial, sentencing and commitment proceedings, depending on the jurisdiction and the type of crime involved (see, e.g., United States v. Fields 2007) (sentencing)). This reluctance extends to the rules governing expert testimony, including the special reliability requirements imposed by the U.S. Supreme Court's holdings in Daubert v. Merrell Dow Pharmaceuticals (1993) and its progeny (see, e.g., General Electric Co. v. Joiner 1997; Kumho Tire v. Carmichael 1999). Further, for better or worse, the standard of proof in pretrial, commitment and sentencing proceedings is seldom the proof beyond a reasonable doubt required at trial (see, e.g., United States v. Comstock 2010) (sexually violent predators). In other countries as well, strictures on evidence and burdens of proof may often be relaxed in the proceedings at issue here.

But neither of these realities should justify basing the deprivations of liberty that routinely occur in these proceedings on immaterial, non‐probative, unhelpful or prejudicial information. If the rules of evidence governing the usefulness of expert testimony apply in civil proceedings involving mere damages (*Daubert* was a tort suit), they should also apply in criminal and quasi‐criminal proceedings that routinely result in pretrial detention, enhanced sentences and indefinite commitment. The usual justification for concluding otherwise is that the decisionmaker in the latter proceedings is almost always a judge or a group of judges, who can be trusted to give evidence its appropriate due. However, the assumption that judges are better than juries at disregarding flawed scientific evidence is highly contestable (Hans 2007, 25). As will become clear in the following discussion, the normal rules applicable to the admissibility of expert testimony, if applied to testimony about risk, should prevent judges from even considering certain types of risk evidence. They would also mandate that evidence that is admissible be subject to limitations both in the way it is presented and the manner in which it is analyzed. Without evidentiary guidelines, judges will be at sea in determining whether to hear evidence about risk and, if they do, what importance to assign to it.

As to the content of those rules, the Federal Rules of Evidence (2023), followed in federal court and most of the states in the United States (Faigman et al. 2024‐2025), provide a good template, one that is arguably consistent with fundamental principles of due process (see *Barefoot v. Estelle* 1983, Blackmun, J., dissenting). Under the federal rules, all evidence, lay or expert, must be “relevant” (Rule 402), meaning that it must have a “tendency to make a fact [of consequence to the proceeding] more or less probable than it would be without the fact” (Rule 401). Additionally, to be admissible, the probative value of this evidence cannot be “substantially outweighed” by its potential for “unfair prejudice, confusing the issues, misleading the jury, undue delay, wasting time, or needlessly presenting cumulative evidence” (Rule 403). When the testimony is an opinion from an expert, the probity of the evidence is subject to even greater scrutiny. Following *Daubert*, Rule 702 states that expert opinion must derive from “scientific, technical or other specialized knowledge” and be based on “sufficient facts or data,” be “the product of reliable principles or methods” and reflect “a reliable application of the principles and methods to the facts of the case.” Further, the rule provides that the testimony must be “helpful to the trier of fact” in deciding the case.

The federal rules also set out a few other stipulations unique to expert testimony. Rule 703 provides that experts may rely on otherwise inadmissible information if “experts in the particular field would reasonably rely” on it but cautions that this information should not be disclosed to the factfinder unless its probative value would “substantially outweigh” its “prejudicial effect.” Rule 704 states that expert testimony may “embrace[]” the “ultimate issue” in the case so long as it is not “about whether the defendant did or did not have a mental state or condition that constitutes an element of the crime charged or of a defense.” Rule 705 states that experts “may be required to disclose” facts or data underlying their opinion on cross‐examination if they are not disclosed on direct, with the caveat from Rule 703 that if the evidence is not otherwise admissible its probative value should substantially outweigh its prejudicial effect. Finally, Rule 706 authorizes the court to appoint its own expert, unless the parties can “show cause” why that should not occur.

All of these rules have an impact on the admissibility and presentation of expert testimony on risk or dangerousness. Those with the most impact are Rule 402's requirement that evidence be “relevant,” Rule 702's twin requirements that expert testimony be reliable and assist the trier of fact, and Rule 403's admonition that even relevant evidence that is reliable and helpful may be excluded on the ground that it will be misunderstood or misused. In the discussion that follows, the import of these rules is reframed to highlight what I regard to be the four central evidentiary inquiries that arise in connection with testimony about risk.

The first two inquiries are derived from the relevance requirement, which I divide into two components. The first is the “materiality” inquiry into whether the evidence is logically related to a proposition in the case, what the *Daubert* Court called “fit” (591; see also Scherr 2003). The second component of relevance is the “probative value” inquiry into whether the evidence is accurate enough that it makes the proposition “more or less probable.” While separating the relevance inquiry into materiality and probative value components is not as common as it once was (see McCormick on Evidence, 1992, 338), it is crucial in this context, because it recognizes that very accurate information about risk may nonetheless not be logically relevant to any proposition in the case and that, conversely, even evidence of risk that clearly addresses a proposition in a case may nonetheless be irrelevant because it is unreliable. This approach also allows integration, through the probative value component, of the reliability inquiry required by Rule 702 and the *Daubert* line of cases.

The third central evidentiary requirement recognized in the federal rules is Rule 702's additional stipulation that expert testimony be “helpful” to the factfinder. This inquiry is separate from that rule's reliability requirement, because even “relevant” (material and probative) expert testimony may be inadmissible if it does not add to what the factfinder could figure out for itself. And finally, even material, probative and helpful testimony might be inadmissible if it is presented in a misleading or confusing manner.

## 3 Materiality (“Fit”)

In the risk assessment setting, the first and conceptually most difficult evidentiary issue is the materiality of opinion testimony. To see why, a brief description of the various types of expert testimony about risk of reoffending is necessary. Such testimony comes in three basic forms: clinical, structured professional judgment, and actuarial (Monahan et al. 2024). Clinical evaluation of risk is the most unstructured. It does not limit the historical, psychological or environmental risk factors to be considered nor the way in which they are combined to reach a conclusion; essentially clinical evaluators may arrive at their opinions any way they see fit. In contrast, structured professional judgment (SPJ) limits the factors to be considered by the evaluator. For instance, one well‐known SPJ approach examines 20 historical, clinical and management factors, no more and no less (K. Douglas and Shaffer 2021; K. S. Douglas and Webster 1999); at the same time, it still leaves up to the evaluator how these factors affect any final opinion. Distinguished from both of these risk assessment techniques is actuarial assessment, which both limits the factors to be considered and structures the weights they are assigned so that a quantified estimate of risk is produced. For instance, one actuarial instrument relies on 12 risk factors, each associated with a particular number of points, which are then added together to obtain a total score that allows comparison to the recidivism rates of individuals with the same or similar scores (Hilton et al. 2021; Harris et al. 2002). Both SPJ and actuarial evaluations rely on “risk assessment instruments,” but only the latter routinely associate numerical probability estimates with an individual.

On the surface, the results from any of these risk assessment techniques may seem material in a proceeding that makes dangerousness a legally relevant criterion. But a more careful analysis would delve into three separate ways risk assessment testimony, even if accurate, might be excluded on logical irrelevance grounds: when it does not address the relevant legal criteria defining risk (legal materiality); when it is based on data that are not generalizable to the case at hand (empirical materiality); and when it is based on risk posed by other people (epistemological materiality). The first two types of materiality are often ignored by the courts; in contrast, the third type needs to be discussed primarily because some courts have *exaggerated* its importance.

### 3.1 Legal Materiality

Of course, one could argue that expert testimony about risk should never be admissible because it is not a legitimate legal criterion, especially at sentencing proceedings, where punishment is the goal and thus backward‐looking retributive factors might be thought to control. I will not address that argument here, although I have elsewhere (Slobogin 2011, 2021). This article will assume that risk is a legally legitimate justification for depriving people of liberty at pretrial, commitment and sentencing proceedings.

But that does not mean that all testimony about risk is legally material. For instance, capital sentencing statutes that make dangerousness an aggravating factor require that the risk posed by the offender involve a “violent act” (see Jurek v. Texas 1976). Commitment under sexual predator statutes requires proof of a risk for committing “predatory acts of sexual violence” (see Kansas v. Hendricks 1997). Many civil commitment statutes require a risk of “substantial bodily harm” (see, e.g., Florida Stat. 2024). Under these types of statutes, a risk assessment that speaks only of “recidivism” or “offending” does not address the legal issue. Actuarial instruments, for instance, are often normed on risk for any type of crime, or on a definition of violent crime that includes simple assaults, which may not meet the “violence” threshold found in these statutes (see, e.g., Hilton et al. 2021, 133). While clinical and SPJ experts can address this concern simply by asserting that the risk they are addressing is the legally relevant one, courts should demand more than bald declarations on this point, a subject to which I will return when discussing the probative value inquiry.

Another legal criterion that risk testimony must address is the duration of the risk. Many instruments provide information about risk over multi‐year followup periods (see, e.g., Hilton et al. 2021, 136; K. Douglas and Shaffer 2021, 274–275). Yet pretrial detention and many sentences are much shorter, making probability estimates from these assessments legally immaterial. Again, given their relative ambiguity in terms of how their conclusions are reached, clinical and SPJ testimony can finesse this issue but should not be allowed to do so.

Finally, both international and domestic law sets out mandates (admittedly, not always followed) that require that the government provide evidence about whether the risk posed by the individual can only be addressed through incapacitation or whether, instead, some less restrictive intervention would achieve its preventive aim (see, e.g., McSherry 2013, 180–182 re international law; Jackson v. Indiana 1972, 738 re American law). Actuarial assessments have difficulty answering this question unless they are focused on risk management as well as risk assessment, which they rarely do; many of them rely primarily on “static” risk factors that are not susceptible to change through treatment. Clinical and SPJ testimony, which are more likely to focus on the types of “dynamic” risk factors that are susceptible to treatment, is much better equipped to address the intervention criterion, but again, should be accompanied by some indicia of reliability on this score, in ways outlined in the discussion of the probative value inquiry.

As should be clear from this brief description, legal materiality depends on the law's definition of dangerousness or risk. Legislatures and courts have done a very poor job of providing such a definition. As I have argued elsewhere, the principle of legality demands that legal entities develop a more finetuned jurisprudence of risk (Slobogin 2021). Just as they have developed a robust legal definition of blameworthiness that delineates the conduct, circumstances and mental states that justify conviction and punishment based on culpability, legal policymakers should explicitly address the outcome, durational and intervention criteria that justify detention based on risk.

Policymakers must also identify the threshold for intervention criterion—that is, the level of risk (e.g., “more likely than not,” “probability”, a particular percentage) that must be met by the relevant standard of proof (e.g., clear and convincing evidence; preponderance of the evidence) before any intervention may take place. That criterion, however, does not affect the materiality inquiry. Say, for instance, that the law requires clear and convincing proof that the individual (or a group with his or her characteristics): (1) more likely than not will (2) commit a violent act (3) within the next 5 years (4) if not imprisoned. Expert testimony must address the last three factors to be material. But it need not assert that the relevant intervention threshold is met. Testimony that an individual poses a low risk might simply mean the government cannot prove its case. Or it might be supplemented by other evidence that allows the government to prevail.

### 3.2 Empirical Materiality

To be material, expert testimony must not only address the relevant legal criteria but also satisfy empirical demands of generalizability. The concern here is whether the sample on which the expert relies in reaching a conclusion is appropriately focused. Some clinical or SPJ experts might find this requirement perplexing, because they view their “sample” as the defendant and the defendant alone; thus, they might respond, of course they have focused on the appropriate sample. This intuition would be correct if all the expert did was offer what I, along with David Faigman and John Monahan, have called “diagnostic” testimony (Faigman et al. 2014, 443–444) and what others have called anamnestic analysis (Heilbrun et al. 2021, 18–19). This is testimony that describes and classifies how a particular person has acted in the past or is acting in the present; for instance, diagnostic testimony could be a statement that a particular defendant has reacted violently to slurs on his manhood, or fondled young boys when alone with them, facts from which the expert then infers that the defendant is likely to do so again under similar circumstances. This type of testimony is akin to character evidence, and it may not require an expert opinion at all.

Most expert testimony about risk, however, is what we called “framework” testimony (Faigman et al. 2014, 421), based on general scientific or specialized knowledge—for instance, testimony that people who have certain biological traits, psychological characteristics or family history (which the defendant has) tend to respond aggressively when their manhood is insulted or to respond sexually when alone with a young boy. When an expert is conveying framework testimony, the “sample” for the opinion is never confined simply to the traits of a particular person. Those characteristics only assume meaning because of the expert's training and experience, the first of which is based on research about other people and the second of which is based on interactions with other individuals, usually those who have been subject to state intervention.

The empirical materiality requirement dictates that this sample “fit” the defendant to the extent feasible. If a psychiatrist has only been trained about and only evaluated people with serious mental illness in emergency commitment settings, or a psychologist relies solely on an SPJ instrument normed on those types of people, their background “sample” is probably not generalizable to, for instance, a sex offender with a personality disorder. Bad sampling fit is even more likely to be apparent (or, put another way, less easily hidden) when the expert relies on an actuarial instrument, the validation sample for which is (or at least can be) well‐known.

In short, to be empirically material, the data or experiences on which the risk assessment is based should be closely aligned to factors relevant to the defendant's case. As Min Yang and his colleagues have stated, samples should be similar to the defendant in terms of “demographic characteristics (e.g., age, gender, socioeconomic status, ethnicity), level and type of past violence (e.g., criminal histories, sexual vs. nonsexual offenders), psychiatric diagnosis (e.g., presence of personality disorder, psychosis), intervention received (e.g., treated v. untreated), the specific criterion being predicted (e.g., violent vs. nonviolent behavior or different types of violent behavior), environmental setting (e.g., clients residing in institutions vs. the community), countries of origin of the research, and so forth (Yang et al. 2010, 741). Obviously, some line‐drawing must occur here; finding a sufficiently large sample with all of the defendant's salient demographic, historical, and environmental features would be impossible. The important point to recognize is that expert testimony based on experience with, or on an actuarial instrument validated on, people who differ significantly from the defendant demographically, geographically, and psychologically should not be considered material, no matter how much experience the expert has or how “accurate” the instrument was shown to be on its validation sample.

In *Wisconsin v.* Loomis (2016), the Wisconsin Supreme Court recognized the importance of empirical materiality but did not require it. *Loomis* considered the permissibility of using the results of an actuarial risk assessment instrument to inform sentencing. The court warned the lower courts that the instrument was not yet validated on a Wisconsin population. But not only did it still permit judges to use the instrument, it upheld the trade secret claim of the instrument's developer, limiting the ability of litigants and others to discover the characteristics of the population on which the instrument had been normed. That conclusion runs directly counter to *Daubert's* fit requirement, which purportedly applies in Wisconsin (Wisc. Stat. § 907.02 2026).

### 3.3 Epistemological Materiality

Many judges have concluded that, even if it is legally and empirically material and highly accurate at what it purports to describe, framework testimony about risk (to the effect that people like the defendant are or are not likely to reoffend) is immaterial, because it is based on information deduced from people other than the defendant. For instance, Justice Coyne of the Minnesota Supreme Court wrote that “statistics concerning the violent behavior of others [are] irrelevant” to a particular defendant's case (In re Linehan 1999, 616). Courts in Virginia, Tennessee and Indiana have excluded testimony about group risk on the ground that it is not “individualized” or “particularized” (Porter v. Commonwealth 2008; United States v. Taylor 2008; Rhodes v. States 2008).

Members of the U.S. Supreme Court, in the recent decision of Diaz v. United States (2024), have waded into the same territory. In that case, the Court held that framework testimony about whether most drug couriers who cross the border know their car is carrying drugs is not “about” the defendant's mental state, and therefore was not excludable under Federal Rule 704's prohibition of expert opinion “about whether a defendant did or did not have a mental state or condition.” That conclusion led the dissent to ask how such testimony is “relevant” in a trial in which the key issue is the *defendant's* knowledge of whether drugs were present (548–549). Thankfully the majority dismissed the dissent's argument and held that the testimony was material (although Justice Jackson's concurring opinion suggested it might have been inadmissible on unreliability grounds) (541–542).

The reason the majority opinion in *Diaz* was correct should be clear from the foregoing discussion. All framework testimony is based on information about people other than the defendant (albeit people who, if the testimony is empirically material, are similar to the defendant). If experts could not rely on statistics or inferences drawn from the study or observation of others, they would not be able to testify about the typical symptoms of schizophrenia, the factors that affect eyewitness accuracy, the characteristics that make people suggestible to interrogation techniques or any other phenomenon expressed in terms of general tendencies, probabilities or likelihoods. Further exposing the illogic of excluding such evidence, in its absence factfinders will engage in their own assumptions about people like the defendant or eyewitnesses, assumptions that, as discussed in connection with the helpfulness requirement, may well rest on misconceptions that experts can correct. Social psychological research has long demonstrated that a crucial way we come to our best (and worst) understandings of unobservable mental states and propensities is through resort to some level of generalization (Jussim 2012). As discussed in more detail in connection with the avoidance of prejudice evidentiary requirement, experts should frame their generalized testimony about risk in a way that does not mislead the factfinder. But such testimony should not be excluded on the ground it is nomothetic (group‐based) in nature.

Thus, the majority in *Diaz* has the better of the argument. The testimony in that case about the typical drug courier's knowledge was not literally “about” the defendant's knowledge. But unless we discard common epistemological techniques, it certainly was relevant to deciding what that knowledge was. While concern about such generalizations is proper, it should not result in wholesale exclusion of all nomothetic information but rather be directed at its empirical materiality (as discussed above), and its probative value, its helpfulness and its potential for misuse (discussed below).

## 4 Probative Value

Ensuring that evidence is material is necessary, but of course not sufficient. When evidence is proffered as expert testimony, American law has insisted on additional indicia of reliability. In some jurisdictions these indicia simply require a showing that the basis of the opinion is accepted in the relevant field (see Frye v. United States 1923). But in most states the testimony must be, in the words of Rule 702, “the product of reliable principles and methods.” The rules use the word “reliable” the way a scientist would use the word “valid” (Daubert 1993, 593–594). According to *Daubert*, reliability (validity) is to be measured by the results of scientific testing, error rates, peer review and, last and apparently of least importance, general acceptance in the relevant field, although other means of verification are also permissible (594–595).

These additional hurdles to the usual demand that evidence be probative are imposed on expert testimony largely out of concern that lay factfinders will not be able to discern its weaknesses but nonetheless give it substantial weight in reaching a decision. As noted earlier, in the proceedings at issue here, a lay jury is almost never involved. But judges who are legally (but often not otherwise) trained may also have difficulty evaluating scientific evidence. The suggestion made here is that, given the complexity of evaluating the validity of risk assessment techniques, this conundrum is best handled in this context by offloading much of the inquiry to entities other than individual judges.

More specifically, the validity of each type of assessment technique would best be decided beforehand, by an independent body that has access to the relevant research. Admittedly, this proposal departs from the traditional process for vetting expert testimony in the United States; the usual assumption, as the Supreme Court itself put it in the context of expert testimony about risk, is that the adversarial system can help the factfinder—judge or jury—“separate the wheat from the chaff” (Barefoot v. Estelle 1983, 899, n. 7). But that is unlikely in this setting, for several reasons. First, attorneys in the types of cases at issue here (usually involving indigent defendants) will not have significant resources to expend on expert consultants, even on the prosecution side. Second, an adversarial process that relies on direct and cross‐examination of opposing experts is not well‐suited to bringing out complicated, nuanced scientific information, especially framework evidence that is based on numerous studies or observations about which even the experts may not be aware. Third, because judges in an adversarial proceeding typically only see two experts, one for each side, they easily succumb to the impression that each side's evidence is plausible even when one of the experts is entirely unrepresentative of the scientific consensus. This phenomenon, which can afflict even highly resourced cases, is referred to as the “99:1 problem:” in an adversarial system, the one maverick expert gets equal time with the expert whose views are backed by the rest of the field (Haw 2012).

Thus, ideally, rather than requiring the presiding judge to engage, in each case, in time‐consuming, often redundant, evaluation of the accuracy of a particular risk assessment technique, a legislative, executive branch or appellate body should do so. Once so vetted, the technique should be considered valid throughout the jurisdiction. Similar proposals have been made by others, mostly on the ground that such a process enhances validity (Neal et al. 2022, 178–179).

Some might object that validity analysis should be left to individual courts because of their familiarity with the case in front of them; indeed, the Supreme Court's decisions after *Daubert* suggest that the trial court should be given substantial deference on this point (General Elec. Co. v. Joiner 1997). Clearly, as I emphasize below (in Section 4.4), whether a valid risk assessment protocol was correctly implemented in an individual case is for the sentencing/commitment court to decide. But, along with Faigman and Monahan, I have also argued that the predicate decision about whether a given scientific method is sufficiently valid to be used at all is analogous to a decision about the validity of a legal doctrine (Faigman et al. 2016). Because scientific principles and methodologies are, like law, generally applicable, they should be subject to jurisdiction‐wide analysis (through legislative enactment, administrative rulemaking, and appellate decisionmaking). Thus, we concluded, once a principle or methodology is found to be valid through that process, it should be treated as precedent and, like precedent, should be reevaluated only when new facts (data) are available. If this approach is considered too antithetical to the adversarial process, however, at the least relevant research about the general validity of a risk assessment technique should be initially presented by an expert appointed by the court, as authorized under federal rule 706, rather than by experts chosen by each party (although the parties could still challenge the court's expert through cross‐examination and rebuttal evidence).

Under either approach, an entity that is independent of the instrument developer, preferably a university or European‐style government institute (Broeders 2003, 248), should aid in assessment of the relevant research. Such research is most likely to exist with SPJ and actuarial instruments. If instead, the expert is relying on clinical prediction that uses no researchable tool, assessing the validity of the technique is much more difficult, because clinicians do not (and usually cannot) follow up their predictions. Thus, at most, studies about clinical risk assessment would have to survey the clinical prediction field as a whole, not the validity of any particular clinician's methodology.

With all of this in mind, information about the following indicia of a risk assessment technique's probative value ought be required: discriminant validity (the ability to distinguish high risk from low risk individuals); calibration validity (the ability to associate groups of individuals with risk probabilities); current validity (the ability to give an accurate account of risk under present conditions); and reliability (the ability to make consistent decisions, both across like cases and within a particular case). Only the last aspect of probative value—coherent decisionmaking in a particular case—should be evaluated on a case‐by‐case basis by the judge.

### 4.1 Discrimination

Given the low base rate of violent crime, if the sole goal is to be correct most of the time, the approach most likely to provide the highest accuracy rate is to predict that no one will recidivate. Depending on the group in question (people subject to civil commitment, insanity acquittees, sex offenders, or the general prison population), that prediction rule would be wrong only between 5% and 20% of the time with respect to violent offending (see, e.g., Langan et al. 2003). But assuming the goal is to identify and incapacitate in some way the highest risk offenders, this blunderbuss mode of decisionmaking would be useless to the legal system. Instead, the proffered risk assessment technique should have some ability to differentiate high and low risk offenders, known as discriminant validity.

Typically, this type of validity is measured by plotting the true positive rate (the rate at which people designated high risk recidivate) against the false positive rate (the rate at which people designated high risk do not recidivate). The value of the “area under the curve” that this plot produces (the AUC) indicates the instrument's discriminant validity (see Mossman 2013). An AUC of 0.5 means the risk assessment technique does no better than chance; in other words, it has no probative value because, to use the definition in Rule 401, it does not make a proposition in the case more or less probable. The AUCs associated with risk assessment techniques, including clinical techniques, generally exceed 0.6 but very few do better than 0.75, a figure indicating that, in studies examining the success rate of the technique, 75% of the time a randomly selected recidivist received a higher score than a randomly selected non‐recidivist (Mossman 2013, 34).

### 4.2 Calibration

In addition to a reasonable AUC value, a technique ought to be able to provide a reasonably accurate estimate of how many people with the defendant's risk factors will recidivate. For instance, one actuarial instrument designates six groups that are associated with ascending levels of recidivism, from around 5% to around 30%; it also provides confidence intervals in connection with each probability estimate, often based on a sample population within the relevant jurisdiction, which improves empirical validity (Arnold Foundation 2024; DeMichele et al. 2018, 523, tbl. 5). Lacking this type of information, a court may find it very difficult to make the normative judgment about the risk level that justifies pretrial detention, commitment or an enhanced sentence.

Calibration is generally only available with actuarial instruments. SPJ assessments are structured with respect to the risk factors considered, but they leave to the evaluator the decision about whether a person poses a high, medium or low risk and what those terms mean (although some studies have attempted to quantify those terms) (see K. Douglas and Shaffer 2021, 257–258; K. S. Douglas and Webster 1999). Similarly, clinical assessments might conclude that a person poses a high or low risk, but either do not define those terms or declare that a person is “likely” or “not likely” to recidivate, based on experience and intuition rather than hard data. The problem in the latter two situations should be apparent from a Rule 702/*Daubert* perspective. Other than general findings suggesting that such predictions are right about as often as they are wrong (see Otto 1994, 63), there are often no “error rates” or other clear indicia of validity that can help other experts or judges assess the probative value of this type of testimony.

### 4.3 Currency

The discriminant and calibration validity of an instrument, even one normed on the jurisdiction in question, can change dramatically over time for a number of reasons. For instance, probability estimates may need adjustment if the make‐up of the population in the jurisdiction changes significantly, the jurisdiction's crime, arrest or conviction rates go up or down substantially, the jurisdiction's policing practices change (with fewer or more people arrested), or the jurisdiction begins implementing innovative alternatives to prison that can reduce risk (see, e.g., Koepke and Robinson 2017). All of these factors can affect base rates for reoffending, so that an instrument validated before the changes take place might estimate a different level of risk than one that was validated after they occur.

Ideally, therefore, periodic audits would be carried out (again, preferably by independent entities) to ensure any risk assessment technique remains sufficiently valid (Hamilton 2020, 95–113; Garrett and Monahan 2020, 487). Periodic auditing is most obviously needed for actuarial instruments, but SPJ and clinical prediction can also be distorted by developments over time. A clinician who evaluates risk based on “years of experience” may be completely out of touch with the newest knowledge about the most potent correlates of violence or the types of people who commit crimes (e.g., women are much more likely to be arrested for violent crime today compared to a decade ago) (Council of Criminal Justice 2025). An SPJ or actuarial instrument validated 15 years ago might pose queries that can lead to invalid conclusions because, for instance, changes in the relevant population have also changed the types of risk factors that are most predictive.

### 4.4 Reliability

As noted above, *Daubert* and the Federal Rules of Evidence often use the term “reliability” in place of validity. But to social scientists, reliability means consistency. In the risk assessment setting, a reliability requirement has three implications. First, risk assessments between cases should be reliable; different experts should reach similar conclusions with respect to people with similar risk factors. This type of reliability is much harder to achieve with clinical and SPJ assessments than with actuarial assessments that are conducted using the same instrument throughout the jurisdiction. However, even with actuarial instruments, inter‐rater agreement can vary significantly, especially if the risk factors (e.g., particular diagnoses, definitions of violent crime) are subject to variable interpretations (Desmarais and Singh 2013).

The second type of reliability focuses on whether the evaluator, in the words of Rule 702, engaged in “a reliable application of the principles and methods to the facts of the case.” This inquiry raises questions such as: Does the defendant have the risk factors (e.g., arrests, convictions, diagnoses) the expert says the defendant has? If an actuarial instrument was used, did the expert score it properly? Did the evaluator make adjustments to the actuarial result and, if so, what were they and why?

This last question raises a third reliability issue that is especially likely to arise when an actuarial approach is used. Because actuarial instruments rely on a limited set of factors, a litigant might object that, in the language of Rule 702, it is not based on “sufficient facts or data”; another way of putting this claim is that actuarial assessments are not sufficiently “individualized.” Reacting to this concern, an evaluator might adjust the probability estimate based on other factors related to the defendant. While in concept this “adjusted actuarial” approach makes sense, research has shown that such adjustments often detract from accuracy, because they are sometimes based on speculation rather than research, or because they “double‐count” risk factors (for instance, criminal history) that are already considered in the algorithm (see, e.g., Angelova et al. 2023; Guay and Parent 2018). Thus, this type of assessment should be permitted only if it can be shown that the proposed adjustments are themselves based on valid research and that they are not derived from factors that were considered (and discarded) during the validation process.

A final observation about evaluating reliability is that, as with validity, determinations of whether inter‐rater reliability for a particular instrument is satisfactory (the first reliability issue described above) are best made by an outside body. As suggested earlier (see the introduction to this section), only the determination of whether the evaluation in the particular case is sufficiently reliable (issues two and three) needs to be, and should be, made by the presiding judge, because that determination is specific to the case at hand. This division of responsibility is also more in tune with the relative competencies of the typical judge and the typical lawyer, who will be much more comfortable double‐checking the evaluation process in a particular case than assessing non‐case‐specific issues such as validity and inter‐rater reliability (see Faigman et al. 2016).

### 4.5 The Comparative Accuracy of Risk and Culpability Assessments

Just as the previous section avoided any conclusion about how much risk justifies imprisonment or other types of interventions, this section has avoided the ultimate issue of how valid and reliable risk assessments must be to meet the requirements of Rule 702 and *Daubert*. Those difficult issues are for the courts and other legal policymakers to decide (although I have made some suggestions on both scores, Slobogin 2021). The only point made here will be a comparative one, focused on the relative accuracy of culpability assessments as they apply at sentencing.

Those who argue that risk should not be relevant at sentencing sometimes compare the difficulties of assessing risk (which should be obvious from the foregoing) with the alleged relative ease of assessing culpability, given the fact that, in the latter case, the relevant harm has been proven beyond a reasonable doubt whereas in the former case it has not yet (and might not ever) occur. This would be an apt point if the comparison were between the accuracy of risk and culpability assessments at the trial stage. But risk is not a pertinent consideration at trial, only at sentencing. So the more appropriate comparator is the accuracy of culpability assessments at sentencing, which often turn not on the harm the defendant caused, but on subtle gradations of mental state at the time of the offense and, in more progressive courts, the biological or environmental constraints on the defendant's ability to choose otherwise (Meixner 2022). To the extent that is so, it is not at all clear that the culpability assessments made at sentencing are any more “accurate” than risk assessments. Indeed, in contrast to risk assessment accuracy, which can be scientifically investigated, the existence, content and impact on behavior of subjective mental states and external factors as they relate to “desert” are not susceptible to measurement (Slobogin 2007, 42–48).

## 5 Helpfulness

Even if expert testimony on risk is material and probative, it may be excluded on the ground that it is not “helpful” to the factfinder as required by Rule 702, because it consists of a commonsense assessment that laypeople are capable of making on their own. If so, the expert testimony becomes superfluous. The most obvious counter to this contention in this setting is that the delineation of risk factors and their relative impact on specific defendants is not something laypeople can easily discern. Additionally, valid risk assessments can overcome the natural, but erroneous, perception that people who have committed violent antisocial acts in the recent past are likely to do so again. In fact, as noted earlier, most such individuals do not reoffend, a point that expert testimony can helpfully bring home in a quantified or quasi‐quantified way. In short, expert testimony about risk can be helpful—or in scientific terms, provide information with incremental validity—to the decisionmaker.

In a 2018 study, Dressel and Farid purported to put the lie to this reasoning by comparing lay decisions about risk to those reached by a well‐known actuarial instrument, the COMPAS (Dressel and Farid 2018). They found that, over 1000 assessments, humans were correct in about 62% of the cases and the COMPAS was correct in 65% of the cases. Several commentators have pointed to this study in arguing that actuarial instruments are no more accurate than lay factfinders, and thus are of no help (see, e.g., Bahl et al. 2024).

The Dressel and Farid study suffered from one significant problem, however. The 20 human subjects were each shown fifty mini‐vignettes that listed only a few features of the defendant, all of which have a robust statistical relationship with reoffending, and were also immediately told, after each decision, whether they were right or wrong. In effect, this methodology turned the humans into algorithms, because they were “trained” to use certain risk factors and not others, and were limited in the factors they could consider. A follow‐up study by Lin et al. found that when lay participants are not provided this type of guidance and feedback, they do much more poorly than an actuarial instrument, even when they are given the base rate of offending among the population in question (Lin et al. 2020). Lin et al. also found that when the information given the humans was “noisier” (i.e., richer in detail than the narrow list of salient factors Dressel and Farid gave their subjects), the humans did barely better than chance, whereas the statistical model had a much higher AUC value. The Lin et al. study suggests that judges, who rarely get feedback about their decisions and virtually always are confronted by considerable noise in pretrial, commitment and sentencing proceedings, are likely to do much worse on their own than when aided by valid expert testimony about risk. That finding is consistent with voluminous research suggesting that mathematical models are more accurate than unstructured intuitive judgments (Meehl 1954; Saks and Kidd 1980‐81; Desmarais et al. 2016).

In short, while the materiality and probative value inquiries will often pose obstacles to the admission of expert testimony on risk, Rule 702's helpfulness requirement usually should not.

## 6 Unfair Prejudice

Material, probative and helpful expert testimony about risk assessment might still be excluded if, to use Rule 403's language, the “danger” that it will be misused is great. This danger might vary depending on the type of risk testimony. Actuarial‐based testimony can be confusing or hard to understand. Clinical and SPJ testimony is less likely to be confusing but, in part for that reason, is more likely to be interpreted by the factfinder to mean that the defendant will (or will not) offend, especially if experts are pushed in a particular direction because of the “adversarial allegiance” phenomenon (Murrie and Boccaccini 2015). This likelihood is a danger because, as even those who rely on clinical and SPJ reasoning acknowledge, “predictions of future offending cannot be achieved, with any degree of confidence, in the individual case” (Cooke and Michie 2010, 272). In other words, experts do not have the scientific or specialized knowledge needed to predict the future of a particular individual with certainty, so any testimony that suggests otherwise could violate Rule 403.

However, the rules of evidence can be deployed to limit these types of dangers. First, experts should not be permitted to assert that a particular defendant will or will not recidivate nor be able to use language (“likely to recidivate”) that cannot be backed up with empirical information. Although Rule 704 permits testimony that embraces the ultimate issue, the commentary to that rule makes clear that, as Rule 702 requires, all expert testimony must stem from “specialized, technical or scientific knowledge”; a definitive statement about recidivism cannot meet this test. Thus, if the expert testimony about risk is based on an actuarial instrument, the opinion should be framed along the following lines: “the defendant shares characteristic with a group, X to Y% of which commit an O type of offense within T period of time, if they are not subject to I types of interventions.” If instead the testimony is based on an SPJ instrument or is clinical in nature, the testimony should avoid any mention of probabilities (because any such mention would be pure guesswork); in particular, contrary to typical practice among some SPJ experts, the evaluator should be prohibited from saying that a person's risk is “high,” “medium,” or “low,” unless these words are empirically tied to probability estimates or at least compared to some identifiable standard (Scurich 2018). Instead, these evaluators should be limited to describing the conditions which, given research or past experience, are most likely to enhance or decrease the chance of offending. Only in these ways can experts avoid misrepresenting the nature of their expertise.

The dissent in *Diaz* claimed that these types of limitations on opinion testimony are disingenuous. Testimony that “most drug couriers know they are carrying drugs,” the dissent argued, is identical to testimony that *Diaz* knew he was carrying drugs. But, in fact, the two statements are quite different. Assuming Diaz refused to describe her mental state, no one other than Diaz can know what Diaz knew about the contents of her car when she was stopped. But we can know, at least in theory, what most drug couriers know. Further, while it is not clear how the expert in *Diaz* knew what most drug couriers know (a probative value issue), if the appropriate research determines that most couriers have this knowledge, communicating that information can be helpful to the factfinder, which otherwise might assume most drugs found in cars at the border were planted there unbeknownst to the courier. Risk assessment testimony, especially when based on actuarial analysis, is even more helpful, because it replaces words like “most,” used in the *Diaz* case, with a probability range that is more concrete and probative.

A second means of insuring against the dangers that expert opinion evidence will be misused is to take seriously Rule 705's provision that the facts underlying an opinion can always be exposed through cross‐examination. In fact, any good expert should describe these facts during *direct* examination, in the course of explaining how the opinion was reached. There should be only two scenarios where that might not happen.

The first is when, under Rule 703, the judge determines that the probative value of the facts underlying the expert opinion does not significantly outweigh their prejudicial effect. (Note that this formulation reverses the usual balancing analysis under Rule 403, which admits evidence unless its relevance is substantially outweighed by its prejudicial impact.) In those jurisdictions that require sentencing entities to consider both retributive and risk factors, Rule 703 could be triggered when, for instance, the factors that suggest decreased risk (e.g., maturity, wealth) also indicate *increased* culpability; risk factors are often orthogonal to factors relevant to desert and thus could undermine accurate consideration of the latter (Stevenson and Slobogin 2018). But this type of conflict should rarely, if ever, lead to exclusion of relevant facts. If both risk and culpability are legitimate sentencing criteria, hiding material and probative facts from the factfinder will ensure flawed reasoning on risk, culpability, or both. In any event, this concern is theoretical in those settings where a judge is the factfinder, since the judge will already know the relevant facts or find out about them if an objection under Rule 703 is made.

Litigants are more likely to make a Rule 703 objection when they believe the expert's description of underlying facts would disclose a trade secret, the type of claim mentioned earlier in connection with the *Loomis* case (see Section 3.2). The contention in such cases is that, when a private company develops a risk assessment instrument, it has a protected intellectual property interest that would be breached if the instrument's risk factors, their weights, and how they are combined are divulged in open court and exposed to its competitors. *Loomis* upheld that argument. But the foregoing discussion about why knowledge of a tool's contents and its validation process is important—both for vetting entities assessing empirical materiality and validity and for judges and lawyers double‐checking the adequacy of expert's evaluation process—counsel the opposite conclusion. Additionally, if the trade secret claim seeks to prevent a prosecution witness from explaining the basis of an opinion, the constitutional right of the accused to confront witnesses may be implicated and should, at the least, be accommodated through *in camera* review, protective orders and the like (Wexler 2018).

## 7 Conclusion

Expert testimony about risk should be legally and empirically material, meet the validity tests of Rule 702 and *Daubert*, add to the factfinder's knowledge about the defendant's risk, and be presented in a manner that minimizes misuse. These inquiries are more easily pursued when the evidence is based on actuarial, rather than clinical or SPJ, evaluations, given the quantified nature of actuarial assessments. But they should apply regardless of the type of testimony proffered.

Ideally legislatures and courts would define precisely the probability, outcome, duration and intervention variables for each type of proceeding in which the risk issue is raised. Risk assessment protocols would be independently evaluated in terms of calibration, discriminant validity, currency and inter‐rater reliability and, if found to be satisfactory in each area, adopted jurisdiction‐wide, subject to periodic auditing. In individual proceedings, judges would only permit experts to use protocols that have been found valid through this process. They would also ensure that the protocol was properly implemented in the case at hand, that the experts' reasoning and factual basis are transparent, and that their testimony about risk avoids misleading, prejudicial characterizations. These rules would promote the most accurate and fair use of expertise about risk.

## Author Contributions

Professor Slobogin is the sole author of this paper.

## Funding

The author has nothing to report.

## Ethics Statement

This paper was written consistent with ethical guidelines.

## Data Availability

The author has nothing to report.
